# The incidence of HIV among women recruited during late pregnancy and followed up for six years after childbirth in Zimbabwe

**DOI:** 10.1186/1471-2458-10-668

**Published:** 2010-11-03

**Authors:** Marshall W Munjoma, Felix G Mhlanga, Munyaradzi P Mapingure, Edith N Kurewa, Grace V Mashavave, Mike Z Chirenje, Simbarashe Rusakaniko, Babill Stray-Pedersen

**Affiliations:** 1 University of Zimbabwe, College of Health Sciences, Department of Obstetrics and Gynaecology, Harare, Zimbabwe; 2 University of Zimbabwe, College of Health Sciences, Department of Community Medicine, Harare, Zimbabwe; 3 University of Oslo, Medical Faculty, Division of Obstetrics and Gynaecology, Rikshospitalet, Oslo, Norway

## Abstract

**Background:**

HIV incidence is a useful tool for improving the targeting of populations for interventions and assessing the effectiveness of prevention strategies. A study in Harare, Zimbabwe reported cumulative incidences of 3.4% (3.0-3.8) and 6.5% (5.7-7.4) among post-partum women followed for 12 and 24 months respectively between 1997 and 2001. According to a Government report on HIV the prevalence of HIV fell from about 30% in 1999 to 14% in 2008. The purpose of this study was to determine the incidence of HIV-1 among women enrolled during late pregnancy and followed for six years after childbirth and to identify risk factors associated with acquisition of HIV.

**Methods:**

HIV-uninfected pregnant women around 36 weeks gestation were enrolled from primary health care clinics in peri-urban settlements around Harare and followed-up for up to six years after childbirth. At every visit a questionnaire was interview-administered to obtain socio-demographic data and sexual history since the previous visit. A genital examination was performed followed by the collection of biological samples.

**Results:**

Of the 552 HIV-uninfected women 444 (80.4%) were seen at least twice during the six years follow-up and 39 acquired HIV, resulting in an incidence (95% CI) of 2.3/100 woman-years-at-risk (wyar) (1.1-4.1). The incidence over the first nine months post-partum was 5.7/100 wyar (3.3-8.1). A greater proportion of teenagers (15.3%) contributed to a high incidence rate of 2.9/100 (0.6-8.7) wyar. In multivariate analysis lower education of participant, RR 2.1 (1.1-4.3) remained significantly associated with HIV acquisition. Other risk factors associated with acquisition of HIV-1 in univariate analysis were young age at sexual debut, RR 2.3, (1.0-5.6) and having children with different fathers, RR 2.7(1.3-5.8). Women that knew that their partners had other sexual partners were about four times more likely to acquire HIV, RR 3.8 (1.3-11.2).

**Conclusion:**

The incidence of HIV was high during the first nine months after childbirth. Time of seroconversion, age and educational level of seroconverter are important factors that must be considered when designing HIV intervention strategies.

## Background

Over 90% of adult HIV infections in sub-Saharan Africa are acquired through sexual contact [1]. Sexual transmission of HIV by older men to young women [2] is the major driving force behind the HIV epidemic in sub-Saharan Africa where 67% (22 million) of the world's HIV-infected population lives, and where approximately 72% of deaths due to AIDS occurred in 2007 [3].

Because of lack of empirical measurements of HIV incidence over an extended period in Zimbabwe, Lopman and Gregson used mortality statistics to back-calculate a peak incidence of 3-6% which they estimated to have occurred between 1988 and 1990 [4], approximately five years after the first HIV case was reported in Zimbabwe. Women followed up post-partum in Harare, Zimbabwe between 1997 and 2001 showed cumulative incidence of 3.4% (3.0-3.8) and 6.5% (5.7-7.4) at 12 and 24 months respectively [5] while an incidence of 4.1% among Zimbabwean women seeking reproductive and general healthcare services followed for 15 to 24 months between 1999 and 2004 was observed [6]. Acute HIV infection in pregnant women and post-partum women is associated with vertical transmission in-utero, during delivery or through breast feeding [7,8]. Consequently, HIV incidence, and not prevalence, is a useful tool for improving the targeting of populations for interventions and assessing the effectiveness of prevention strategies. Incidence can also be used for monitoring the HIV epidemic.

Meanwhile the prevalence of HIV-1 in Zimbabwe has declined steadily from 29.3% in 1999 to 26.5% in 2001, 23.2% in 2003, 19.4% in 2005 and finally to 15.7% in 2007 [9]. This steady decline is primarily due to changes in sexual behavior [10,11]. There are few current reports on HIV incidence in Zimbabwe, particularly in the period after 2004. The purpose of this study therefore is to determine the incidence of HIV-1 among women enrolled late in pregnancy and followed for six years after childbirth and to identify risk factors associated with acquisition of HIV.

## Methods

### Study design and population

The study participants were HIV-uninfected women who were enrolled around 36 weeks gestation. The women were attending routine antenatal clinics at three randomly selected primary health care clinics around Harare between April 2002 and September 2004 and followed up until August 2008. They were followed up at six weeks, four months, nine months and every six months up to six years after childbirth. At enrolment the women were tested and counseled for HIV as part of a national PMTCT program. A standardized questionnaire was interview-administered to capture socio-demographic data, reproductive history and sexual behavior in order to identify risk factors for recent HIV infection. A physical examination and a genital examination were done. Two high vaginal swabs for testing *Trichomonas vaginalis*, yeasts and clue cells in wet mounts were collected. Blood samples were tested for syphilis and HSV-2 infections at enrolment. Participants with symptoms of curable STIs were treated and were also requested to bring their male partners for counseling, testing and treatment of the STIs. The participants were pre-test and post-test counseled for HIV by trained counselors.

At every follow-up visit another structured questionnaire was interview-administered to capture sexual behavior relating to the period since the previous visit. Blood samples were collected at follow-up visits for testing antibodies to HIV-1.

### Laboratory Methods

Parallel testing for HIV infection was performed at enrolment on all the participants on two separate occasions. Firstly they were tested by a nurse at the primary health care clinic as required by the National PMTCT program and secondly by a laboratory technician at the laboratory. The HIV rapid tests used on both occasions were Determine, (Abbott Diagnostics, Wiesbaden, Germany) and Oraquick, (Orasure, Bethlehem, PA, USA). The main reason for re-testing in the laboratory was to make sure that everybody was truly negative at enrolment. Only participants that were negative by both test providers (nurse and laboratory technician) were invited to participate. Syphilis infection was screened using a rapid plasma reagin (RPR) test and all reactive samples confirmed by Treponema Palidum Hemaglutination Assay (TPHA), both tests manufactured by Randox Laboratories Ltd., Ardmore, UK. A wet mount from freshly collected vaginal fluid was examined microscopically for *Trichomonas vaginalis *and yeast cells. Whiff test, clue cells and absence/presence of lactobacilli were also evaluated in the laboratory and used as an estimation of bacterial vaginosis due to the absence of pH measurements and vaginal discharge which form part of Amsel criteria.

Parallel testing with Determine and Oraquick rapid HIV test kits was also employed at every follow-up visit and samples that were reactive on at least one of the rapid HIV tests were confirmed with Western Blot (Biorad Laboratories, Marnes La Coquette, France), according to the algorithm adopted for establishing new HIV infections in this protocol. Furthermore, all babies whose mothers had seroconverted at six weeks were tested for HIV infection using Amplicor HIV-1 DNA Test, version 1.5, (Roche Molecular Systems, Branchburg, NJ, USA) to determine antepartum or intrapartum transmission of HIV. All laboratory tests were done in the Department of Obstetrics and Gynecology in the College of Health Sciences in Harare, Zimbabwe.

### Ethical considerations

The study was approved by both the Medical Research Council of Zimbabwe and the Norwegian Ethical Committee. All the participants provided written informed consent prior to study participation.

### Data analysis

Data were analysed using SPSS version 16.0 (SPSS, IL, USA) and STATA version 10 (Tx, USA) and summary statistics were used to describe the sample population. Relative risks (RR), both univariate and multivariate together with 95% confidence interval (CI) were calculated. In multivariate analysis risk factors that had a p value < 0.25 in univariate analysis as well as other factors that were known to be associated with seroconversion were included. Incidence rate was calculated using survival analysis method and expressed as woman-years at risk (wyar).

## Results

A total of 594 pregnant women were HIV-uninfected according to the national PMTCT program and 571 were HIV-uninfected according to tests done in the laboratory. Altogether 552 participants were HIV-uninfected by both the PMTCT national program and the laboratory. At baseline the pregnant women were aged between 15 and 42 years with a median age of 22 years, had median life-time pregnancies of two and median number of living children of one. The majority of the pregnant women (63.8%) were below 25 years of age at the time of enrolment. About 95% of the women were married or living with a partner, 2.9% single while 2.6% divorced, separated or widowed. Only 6.4% reported being formally employed.

The prevalence for HSV-2, *Trichomonas vaginalis*, yeasts and syphilis at baseline was 34.2%, 9.4%, 38.0% and 0.4% respectively. Other parameters examined were whiff test 33.2%, clue cells 43.8% and absence of lactobacilli 40.8%. Over 80% of the participants harbored at least two of the above infections and/or conditions.

Over 80% (444) of the women attended a minimum of two visits during the follow-up period resulting in 1687.9 woman-years at risk (wyar) of follow up, with a mean follow-up period of 38.2 months. A total of 39 participants acquired HIV over the six-year follow-up period. The age range and average age at acquisition of HIV was 17-41 years and 24.8 years respectively. Of note were four pregnant teenagers who were still teenagers when they acquired HIV within one year of enrollment. The overall incidence during the entire period was 2.3/100 wyar, 95% CI 1.1-4.1 (Table 1). There was a relatively high incidence of 4.4/100 wyar 95% CI 2.6-6.7 which occurred between enrolment and six weeks after childbirth as a result of four mothers who acquired HIV but however none of these four mothers transmitted the virus to their babies in utero or during childbirth. The highest incidence of 7.5/100 wyar 95% CI 4.8-10.9 was observed between four and nine months after childbirth (Table 1). A summary of the incidence after delivery shows that 17 of the 39 seroconverters (43.6%) acquired HIV during the first 9 months after childbirth resulting in an incidence of 5.7/100 wyar 95% CI 3.3-8.1. Of these 17 seroconverters at nine months 15 (88.2%) were aged below 25 years at the time of enrolment. Also, among the different age groups at enrolment teenagers had the highest incidence of 2.9/100 wyar 95% CI 0.6-8.7 followed by the 20 to 24 year age group with an incidence of 1.7/100 wyar 95% CI 0.3-4.7 (Table 2).

**Table 1:** HIV incidence rates at different time points between delivery and 72 months after childbirth.

	No. of participants			
Observational period	Tested	seroconverted	Total person years at risk (wyar)	Incidence rate per 100 wyar (95% CI)
Enrolment to 6 weeks pp	436	4	90.8	4.4 (2.6-6.7)
>6 wks to 4 mths pp	352	3	73.3	4.1 (2.2-6.6)
>4 mths to 9 mths pp	321	10	133.8	7.5 (4.8-10.9)
>9 mths to 72 mths pp	269	22	1390.0	1.6 (0.4-3.7)
Overall		39	1687.9	2.3 (1.1-4.1)

wyar = woman-years-at-risk, pp = postpartum.

**Table 2:** HIV incidence during 6 years according to age groups at recruitment

Age group in years	n	Seroconverters n (%)	Incidence rate (95% CI)
<20	98	15 (15.3)	2.9 (0.6-8.7)
20 - 25	184	16 (8.7)	1.7 (0.3-4.7)
25 - 29	101	5 (5.0)	1.0 (0.03-5.4)
30 - 34	42	1 (2.4)	0.9(.001-8.4)
>34	17	2 (11.8)	1.1(.001-19.5)
15 - 42	442*	39 (8.8)	2.3(1.1-4.1)

wyar = woman-years-at-risk * No date of birth provided on two participants.

Unadjusted relative risk (RR) with significant associations of acquisition of HIV-1 were low education of participant, RR 2.1, (1.1-4.3), young age at sexual debut, RR 2.3, (1.0-5.6), number of life-time partners >2, RR 2.1, (1.1-4.1), siblings with different fathers, RR 2.7, (1.3 - 5.8) and genital itching RR 1.8, (1.0-3.4). In multivariate analysis having low education remained significantly associated with acquisition of HIV-1. Also, those that reported having been sexually abused, adjusted relative risk (ARR) 3.1(1.2-8.0), and syphilis ARR 8.5 (1.4-50.4) were significantly associated with acquisition of HIV-1 (Table 3).

**Table 3:** Adjusted and unadjusted relative risk for acquisition of HIV among pregnant women.

Variable	No. tested	No. HIV positive (%)	Unadjusted RR (95% CI)	Adjusted RR (95% CI)
*Age of participant in years*				
35-42	17	1(5.8)	Reference	Reference
30-34	42	2(4.8)	0.8(0.1-8.4)	1.0(0.1-9.9)
25-29	101	6(5.9)	1.0(0.1-7.9)	0.9(0.1-8.6)
20-24	184	15(8.2)	1.4(0.2-9.9)	1.1(0.1-9.2)
15-19	98	15(15.3)	2.6(0.4-18.5)	2.2(0.3-18.2)
*Marital status*				
Married	428	36(8.4)	Referent	Referent
Single	13	3(23.1)	0.3(0.1-1.2)	0.4(0.1-1.7)
*Polygamous marriage*				
No	396	30(7.6)	Referent	Referent
Yes	29	5(17.2)	2.3(0.9-5.9)	2.6(0.8-8.5)
*Have you been sexually abused?*				
No	418	34(8.1)	Referent	Referent
Yes	22	4(8.2)	2.2(0.8-6.3)	3.1(1.2-8.0)
*Education level of participant.*				
>primary	375	28(7.5)	Referent	Referent
<primary	69	11(15.9)	2.1(1.1-4.3)	2.5(1.1-5.3)
*Is partner a frequent traveler?*				
No	252	17(6.7)	Referent	Referent
Yes	174	18(10.3)	1.5(0.8-3.0)	0.9(0.4-2.0)
*Ever used intravaginal herbs?*				
No	380	33(8.7)	Referent	Referent
Yes	61	5(8.2)	0.9(0.4-2.4)	0.7(0.2-2.3)
*Age at sexual debut*				
≥16	131	6(4.6)	Referent	Referent
<16	308	33(10.7)	2.3(1.0-5.6)	1.9(0.5-7.6)
*Used condom at sexual debut?*				
Yes	45	6(13.3)	Referent	Referent
No	393	32(8.1)	1.6(0.7-3.9)	1.5(0.5-4.7)
*Ever used a condom?*				
No	246	22(8.9)	Referent	Referent
Yes	191	17(8.9)	1.0(0.5-1.9)	1.2(0.5-3.1)
*Casual partners in past 12 months*				
No	433	36(8.3)	Referent	Referent
Yes	9	2(22.2)	2.7(0.6-11.1)	2.0(0.6-7.0)
*No. of lifetime sexual partners*				
<2	350	25(7.1)	Referent	Referent
≥2	93	14(15.0)	2.1(1.1-4.1)	0.8(0.3-2.2)
*Siblings have same father?*				
Yes	370	27(7.3)	Referent	Referent
No	45	9(20.0)	2.7(1.3-5.8)	1.8(0.6-4.9)
*Do you have genital itching?*				
No	291	20(6.9)	Referent	Referent
Yes	151	19812.6)	1.8(1.0-3.4)	0.8(0.4-2.0)
*Laboratory-diagnosed syphilis*				
Negative	403	35(8.7)	Referent	Referent
Positive	2	1(50.0)	5.8(0.8-42.0)	8.5(1.4-50.4)
*Clinical genital warts*				
Absent	381	33(8.7)	Referent	Referent
Present	18	4(22.2)	2.7(0.9-7.2)	1.2(0.9-1.5)

General knowledge about HIV and how it is transmitted or acquired were also assessed (Table 4) and only those participants that reported knowing that their partners had other sexual partners were at risk of acquiring HIV-1, RR 3.8(1.3-11.2). Although there is more general knowledge and positive attitudes regarding HIV issues amongst the study participants it must be noted that about 90% of both non-seroconverters (349/394) and seroconverters (29/32) did not believe that abstinence was protective against acquisition of HIV-1.

**Table 4:** Relative risk derived from knowledge factors associated with acquisition of HIV amongst pregnant women

Variable	No. tested	No. HIV positive (%)	RR(95% CI)
*How do you rate your chances of infection?*			
No risk	92	10 (10.9)	Referent
Small risk	78	5 (6.4)	0.6(0.2-1.7)
Moderate risk	67	4 (6.0)	0.5(0.2-1.7
High risk	101	9 (8.9)	0.8(0.3-1.9)
Don't know	101	11 (10.9)	1.0(0.4-2.3)
*Why do you think you have no risk?*			
I am married	34	4 (11.8)	Referent
I have one sexual partner	20	2 (10.0)	0.8(0.2-4.3)
My partner is faithful	36	5 (13.9)	1.2(0.3-4.0)
I use condoms regularly*	2	0 (0)	0.2(0.03-2.3)
Others	31	1 (3.2)	0.8(0.1-5.8)
Not applicable	12	1 (8.3)	
*Why do you think you are at risk?*			
Partner cannot be trusted	156	9 (5.8)	Referent
Partner has other sexual partners	18	4 (22.2)	3.8(1.3-11.2)
I have other sexual partners	1	0 (0)	Incalculable
We do not use condoms	30	2 (6.7)	1.2(0.3-5.1)
Others	25	1 (4.0)	0.7(0.1-5.3)
Not applicable	18	1 (5.6)	1.0(0.1-7.2)
*Can consistent condom use prevent STI/HIV?*			
Yes	339	28 (8.3)	Referent
No	56	4 (7.1)	1.2(0.4-3.2)
*Abstain as prevention of STI and HIV?*			
Yes	45	3 (6.7)	Referent
No	349	29 (8.3)	0.8(0.3-2.5)
*Do you know someone infected with HIV?*			
Yes	146	8 (5.5)	Referent
No	296	31	0.5(0.2-1.1)
*Do HIV-infected people always show signs?*			
No (Can look healthy)	287	23 (8.0)	Referent
Yes (Always show signs)	124	12 (9.7)	0.8(0.4-26.8)
Do not know	27	4 (14.8)	1.5(0.5-4.4)
*Are healthy-looking infected men infectious?*			
Yes	408	34 (8.3)	Referent
No	15	1 (6.7)	1.3(0.2-8.6)
Do not know	18	4 (22.2)	3.3(0.4-26.8)
*Can a woman transmit HIV to her baby?*			
Yes	415	35 (8.4)	Referent
No	1	0	Incalculable
Do not know	17	4 (19.0)	2.3(0.9-5.8)
*Can you discuss AIDS with a family member?*			
Yes	335	30 (9.0)	Referent
No	108	9 (8.3)	1.1(0.5-2.2)
*Can you discuss AIDS with your spouse?*			
Yes	386	32 (8.3)	Referent
No	56	7 (12.3)	0.7(0.3-1.4)
*Can you discuss AIDS with your friends?*			
Yes	317	28 (8.8)	Referent
No	125	11	1.0 (0.5-2.0)

## Discussion

Our study which was conducted between 2002 and 2008, showed an incidence of 5.7/100 wyar (3.3-8.1) at nine months post-partum and an overall HIV incidence of 2.3/100 wyar (95% CI 1.1-4.1) six years post-partum. However a similar study conducted in Harare between November 1997 and January 2001 showed cumulative incidences of 3.4% (3.0-3.8) and 6.5% (5.7-7.4) amongst women followed up for 12 and 24 months post-partum respectively [5]. By comparing our incidence of 5.7/100 wyar (3.3-8.1) at nine months to that of the previous study of 3.4% (3.0-3.8) at 12 months we can see clearly from the confidence intervals that there are no statistically significant differences between the two estimates, indicating that in this study we are unable to provide evidence of a declining incidence. However the overall incidence of 2.3/100 over the entire six years follow-up period appears to indicate a general decline although this can be attributed to following up the same group over a long period. As HIV-1 prevalence continues to decline in Zimbabwe there is need to develop and implement effective local strategies that will result in a reduction of the incidence of HIV.

During the early days of the HIV epidemic acquisition of HIV infection was associated with an increased level of education and high socioeconomic position [12]. Twenty-five years into the epidemic this study confirms that low education among women is now significantly associated with acquisition of HIV-1. In Tanzania this reversal of risk of acquisition of HIV from educated to non-educated was noticed between 1991 and 2005 [13]. If all children, particularly girls, are educated beyond primary school the end-result may lead to a reduction of the incidence of HIV [13]. We also note an association between acquisition of HIV and sexual abuse among women but suggest that this needs a qualitative approach to reveal pertinent aspects of the abuse.

We also showed that women that have children fathered by different men are about three times more likely to acquire HIV compared to women whose children have the same father (Table 3). Having children with different men reflects a high level of risky behavior as it shows a tendency of having unprotected sex with different men. Since about 70% of the participants were below 25 years of age at enrolment such risky behavior could be a result of inability to meet reproductive health needs after sexual debut [14], as corroborated by the high rate of co-infections in this population. Over 80% of the women had at least two sexually transmitted infections/conditions. STIs are important in that most HIV infections occur in the presence of other STIs [15-18].

The earliest HIV-1 seroconversions were noticed at the six weeks' visit after childbirth (Table 1). Because HIV-1 antibodies can take between three and six weeks to appear in detectable amounts in peripheral blood [19] the participants that had detectable HIV antibodies at six weeks could have been actually infected before enrollment but had undetectable amounts of antibodies at the time of screening which was on average four weeks before childbirth. The mothers may possibly have been in the acute phase of infection around the time of delivery and yet they did not receive nevirapine for PMTCT and were therefore at risk of transmitting the virus to their babies antepartum, intrapartum or during breastfeeding [7,20]. Furthermore, because of its traumatic nature, vaginal delivery often causes microscopic ulcerations [21] which may increase HIV portals of entry/exit. It is important therefore to emphasize condom use in preventing transmission of STIs including HIV-1 during pregnancy and after delivery. Also there is need for use of simple and affordable point-of-care tests that can detect primary infections late in pregnancy in order to reduce the risk of mother to child transmission.

Pregnancy, whether intended or unintended, is an outcome of unprotected sex. An incidence rate of 5.7/100 wyar within nine months of delivery demonstrates that pregnancy is one of the major events following which HIV seroconversion can occur. HIV acquisition may be high during breastfeeding since it is considered a safe period where no other contraceptive methods (including condoms) need to be used. However we hope that the opt-out testing scheme recently introduced in antenatal clinics will increase the number of pregnant women with knowledge of their HIV status [22] and thereby help reduce transmission of HIV.

About 50% (18/39) of the seroconverters were aged 20 years and below at enrollment but of note are the four that were still teenagers when they acquired HIV. These four teenagers initiated sex, became pregnant, acquired HIV (and possibly other STIs) and gave birth, all in a short period before becoming mature adults. Better health for young women and men is most likely to be achieved if youths delay their sexual debut until they are physically and mentally mature to begin their married life [23]. Instead, the young women have relationships with older partners and the age difference between them and their partners is a risk factor for acquisition of HIV [24]. Furthermore, women that knew that their partners had other sexual partners were also at a greater risk of acquiring HIV demonstrating how powerless women are in negotiating safe sex (Table 4). It is therefore imperative for preventive strategies to engage men, especially when almost 100% of married women report having sex with their spouses only [25]. A high incidence in young adults needs to be addressed if a future AIDS-free generation is to be achieved and this has to be addressed from the perspectives of both the young woman and the older partner. Men can be involved by setting up mobile men-friendly reproductive health clinics in industrial areas where they are free to attend to get treatment and/or counseling. The prevalence of HIV at baseline in this cohort was 25.6%. The prevalence has continued to decline to reach 15.1% in 2008 [3] and this decline is attributed primarily to behavior change [11] yet our study shows that about 90% of both the seroconverters and non-seroconverters in this study do not believe that abstinence protects against HIV (Table 4) even though abstinence, be faithful and condom use (ABC) has been for a long time a cornerstone of HIV prevention strategies.

Following up the same group of participants for a long time has its limitations which may demand careful interpretation of the data. Firstly, there may be a systematic selection of those participants that are less likely to seroconvert thereby leading to a possible underestimation of the true incidence. Secondly, as young people get older a high risk population is removed from the cohort with no replacement and this may again lead to the underestimation of HIV incidence. In this study the youngest woman at enrolment (15 years) was 21 years at the end of the study. Thirdly, the same participants may become targets of several other HIV prevention studies thereby resulting in an apparent reduction of incidence. Finally, the introduction of VCT facilities and the "opt out" option may result in more participants knowing their HIV status thus causing people to less likely acquire HIV [22].

## Conclusion

The incidence of HIV in our study, particularly at nine months, is not significantly different from the figures reported in previous studies. Time of seroconversion, age and educational level of seroconverter are very important factors that must be considered when designing HIV intervention strategies. With more HIV-infected people living longer due to ARVs it is imperative to continue to educate people, particularly young people, on the importance of condom use to prevent HIV transmission with a view to reduce the incidence.

## Competing interests

The authors declare that they have no competing interests.

## Authors' contributions

MM and FM were involved in designing, concepting and drafting of the manuscript. EK assisted in collecting data and revision of the initial draft. PM and SR were involved in the statistical analysis and interpretation of data. MC was involved in concepting, analysis and interpretation of data. GM was involved in acquisition and interpretation of laboratory data as well as revision of the first draft while BS-P was involved in concepting, designing, analysis and drafting of manuscript. All the authors read and approved the final manuscript.

## Pre-publication history

The pre-publication history for this paper can be accessed here:

http://www.biomedcentral.com/1471-2458/10/668/prepub
